# Microstructural abnormalities in callosal fibers and their relationship with cognitive function in schizophrenia: A tract‐specific analysis study

**DOI:** 10.1002/brb3.1357

**Published:** 2019-07-08

**Authors:** Yuji Ohoshi, Shun Takahashi, Shinichi Yamada, Takuya Ishida, Kumi Tsuda, Tomikimi Tsuji, Masaki Terada, Kazuhiro Shinosaki, Satoshi Ukai

**Affiliations:** ^1^ Department of Neuropsychiatry Wakayama Medical University Wakayama Japan; ^2^ Wakayama‐Minami Radiology Clinic Wakayama Japan; ^3^ Asakayama General Hospital Osaka Japan

**Keywords:** cognition, corpus callosum, diffusion tensor imaging, magnetic resonance imaging, schizophrenia

## Abstract

**Introduction:**

The corpus callosum serves the essential role of relaying cognitive information between the homologous regions in the left and the right hemispheres of the brain. Cognitive impairment is a core dysfunction of schizophrenia, but much of its pathophysiology is unknown. The aim of this study was to elucidate the association between microstructural abnormalities of the corpus callosum and cognitive dysfunction in schizophrenia.

**Methods:**

We examined stepwise multiple regression analysis to investigate the relationship of the fractional anisotropy (FA) of callosal fibers in each segment with *z*‐scores of each brief assessment of cognition in schizophrenia subtest and cognitive composite score in all subjects (19 patients with schizophrenia [SZ group] and 19 healthy controls [HC group]). Callosal fibers were separated into seven segments based on their cortical projection using tract‐specific analysis of diffusion tensor imaging.

**Results:**

The FA of callosal fibers in the temporal segment was significantly associated with *z*‐scores of token motor test, Tower of London test, and the composite score. In the SZ group, the FA of callosal fibers in the temporal segment was significantly associated with the *z*‐score of the Tower of London test. In addition, the FA of callosal fibers in temporal segment showed significant negative association with the positive and negative syndrome scale negative score in the SZ group. Compared to the HC group, the FA in temporal segment was significantly decreased in the SZ group.

**Conclusion:**

Our results suggest that microstructural abnormalities in the callosal white matter fibers connecting bilateral temporal lobe cortices contribute to poor executive function and severe negative symptom in patients with schizophrenia.

## 1 INTRODUCTION

The disconnection hypothesis in schizophrenia (Friston, 1999) is supported by repeated reports of abnormalities of white matter (WM) fibers that connect brain regions (Kubicki & Shenton, 2014; Samartzis, Dima, Fusar‐Poli, & Kyriakopoulos, 2014; Wheeler & Voineskos, 2014). The corpus callosum (CC), the largest commissural fiber bundle in the brain, connects the left and the right hemispheres and serves an essential role of relaying sensory, motor, and cognitive information between the homologous regions (Huang et al., 2005; Ribolsi, Daskalakis, Siracusano, & Koch, 2014). Many studies have investigated abnormalities of the CC in schizophrenia with an aim of studying the disconnection between the two hemispheres (Isobe et al., 2016; Ribolsi et al., 2014).

Advances in diffusion tensor imaging (DTI) have enabled capture of microstructural WM abnormality. Techniques to build three‐dimensional fiber tracts based on DTI provide opportunities to investigate how specific fiber tracts may affect disorders, by visualizing the trajectories of specific WM fiber bundles and by quantitatively characterizing each fiber tract (Lee et al., 2005; Wakana et al., 2007).

Cognitive impairment is a core dysfunction of schizophrenia that is associated with functional prognosis (Green & Harvey, 2014), but much of its pathophysiology is unknown. Cognitive performance is strongly associated with communication between multiple brain regions (Fox et al., 2005). Development of CC in childhood is correlated with intelligence, processing speed, and problem‐solving ability, and is thought to play a fundamental role in cognitive functioning (Hinkley et al., 2012). However, few studies have performed detailed investigation of the relationship between microstructural abnormalities of CC and cognitive functioning in schizophrenia.

In the current study, we examined the association between microstructural abnormalities of CC fibers and cognitive dysfunction in schizophrenia by segmenting the CC fibers based on their cortical projection regions using DTI tract‐specific analysis (TSA). We hypothesized that patients with schizophrenia show microstructural abnormalities in CC fibers and these abnormalities are related to their cognitive impairment.

## 2 METHODS

### 2.1 Subjects

The subjects were 19 patients with schizophrenia (SZ group) and 19 healthy controls (HC group; Table 1). The subjects were diagnosed by two independent well‐trained psychiatrists based on the Diagnostic and Statistical Manual of Mental Disorders, Fourth Edition (APA, 1994), and were recruited from Wakayama Medical University Hospital. Patients with comorbid psychiatric, neurological, or medical illness, or those with substance or alcohol abuse were excluded from the study. All patients were on antipsychotic medication. Equivalent doses of antipsychotics were calculated using the equivalent conversion table originally reported by Inagaki and Inada (Inada & Inagaki, 2015). This study was approved by the Ethics Committee of Wakayama Medical University, and written informed consent was obtained from all subjects.

**Table 1:** Demographic and clinical characteristics

	HC group (*n* = 19)		SZ group (*n* = 19)		Statistics	
	Mean ± *SD*	Range	Mean ± *SD*	Range		*p*
Gender, male/female^a^, *n*	7/12		9/10		*χ* ^2^ = 0.43	0.45
Age^b^	41.89 ± 10.25	30–60	44.16 ± 7.98	34–60	*t* = −0.76	0.74
Duration of illness, years			18.58 ± 10.12			
PANSS positive			14.05 ± 5.78			
PANSS negative			18.16 ± 5.70			
PANSS general psychopathology			32.53 ± 10.36			
PANSS total			64.74 ± 20.11			
Medication, CPZ equivalent (mg/day)			642.26 ± 330.66			
Verbal memory^b^, *z*‐score	−0.04 ± 0.12		−2.17 ± 1.30		*t* = 5.25	0.00
Digit sequencing^b^, *z*‐score	0.21 ± 0.94		−1.52 ± 0.95		*t* = 5.63	0.00
Token motor task^b^, *z*‐score	0.19 ± 0.90		−1.88 ± 1.62		*t* = 4.89	0.00
Verbal fluency^b^, *z*‐score	0.37 ± 1.00		−1.31 ± 1.11		*t* = 4.88	0.00
Symbol coding task^b^, *z*‐score	0.92 ± 1.10		−1.86 ± 1.97		*t* = 5.38	0.00
Tower of London^b^, *z*‐score	0.04 ± 0.94		−1.72 ± 2.19		*t* = 3.22	0.00
Composite score^b^, *z*‐score	0.28 ± 0.78		−1.74 ± 1.17		*t* = 6.25	0.00

Abbreviations: CPZ, chlorpromazine; HC, healthy controls; *n*, number; PANSS, Positive and Negative Syndrome Scale; *SD*, standard deviation; SZ, schizophrenia.

a Chi‐square test.

b Independent‐samples *t* test.

### 2.2 Neuropsychological measurements

The severity of clinical symptoms was assessed using the Positive and Negative Syndrome Scale (PANSS). Neurocognitive function was tested by experienced psychologists using the Brief Assessment of Cognition in Schizophrenia (BACS) Japanese version (Kaneda et al., 2007), which is widely used in Japan (Ikebuchi et al., 2017; Itakura et al., 2017; Satogami, Takahashi, Yamada, Ukai, & Shinosaki, 2017; Sawada et al., 2017; Takahashi et al., 2013). This battery includes six subtests: list learning test (verbal memory), digit sequencing test (working memory), token motor test, verbal fluency test, symbol coding test (attention), and the Tower of London test (executive function). In the BACS, *z*‐scores were calculated for each subcomponent score using means and standard deviations based on the dataset of healthy Japanese populations (Kaneda et al., 2013). The composite score was calculated by averaging all *z*‐scores of six subcomponents.

### 2.3 MRI data acquisition

We acquired anatomical MRI and DTI data on a 3.0T MR scanner (Achieva TX 3.0T; Philips Medical Systems) using a 32‐element sensitivity‐encoding head coil. A 3D fast field echo T1‐weighted sequence was used for anatomical MRI (TR/TE = 7.0/3.3 ms, FOV = 220 mm, 210 slices, acquisition voxel size = 0.86 × 0.86 × 0.9 mm, and a slice thickness = 0.9 mm). DTI images were acquired using a single‐shot spin‐echo echoplanar imaging diffusion sequence with fifty‐five 2.5‐mm slices (no interslice gap), TR/TE = 6,421/69 ms, FOV = 224 mm, acquisition voxel size = 2.0 × 2.0 × 2.5 mm, and 2b values of 0 and 1,000, 15 directions.

### 2.4 DTI data processing

**Figure 1:**
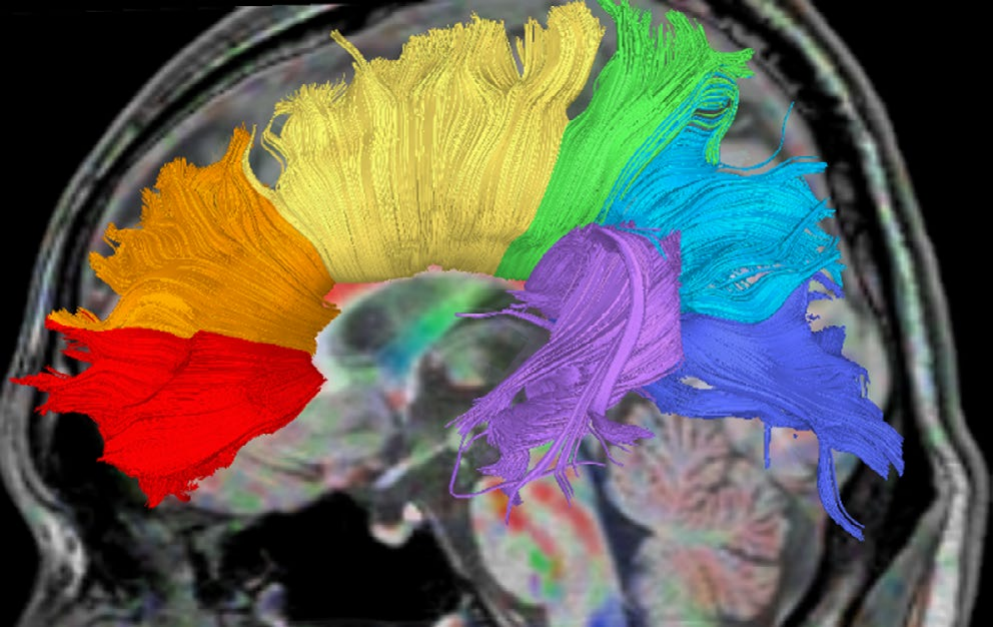
Segmentation of corpus callosum by tractography. The corpus callosum was subdivided into seven separate segments using a two‐regions‐of‐interest (ROIs) fiber tracking approach in accordance with a determined rule and specific anatomical landmarks. The seven segments are, in order from most front to most back, as follows: orbital frontal (OF), anterior frontal (AF), superior frontal (SF), superior parietal (SP), posterior parietal (PP), temporal (Temp), and occipital (Occ)

**Figure 2:**
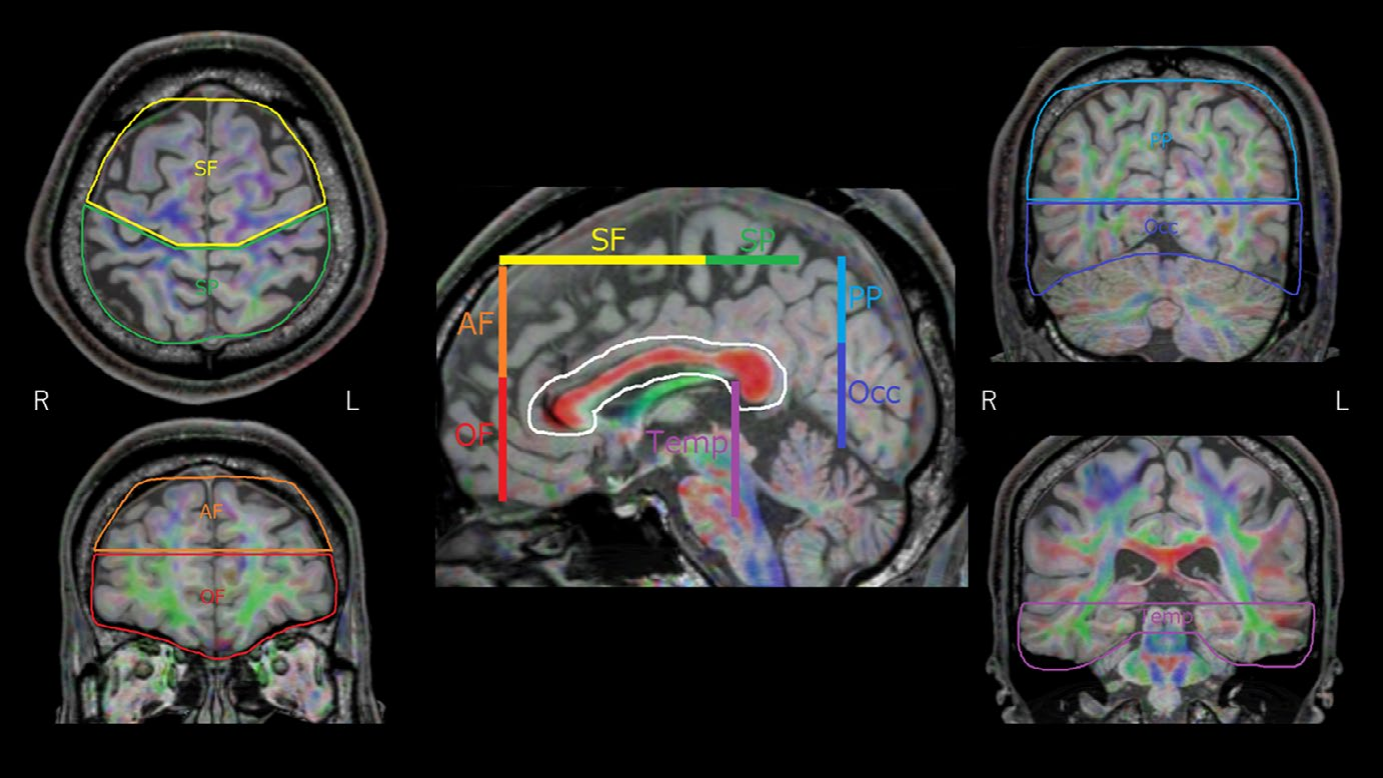
Locations of secondary regions‐of‐interest (ROI) to separate fibers projecting to different cortical areas

Some DTI‐derived data, such as fractional anisotropy (FA) and the apparent diffusion coefficient (ADC), provide information on WM diffusion (Basser & Pierpaoli, 1996). FA is a composite measure of three eigenvalues (*λ*_1_, *λ*_2_, and *λ*_3_). *λ*_1_, the largest in three, which is called the axial diffusivity (AD), is the component parallel to, and *λ*_2_ and *λ*_3_, whose average is called the radial diffusivity (RD), are components perpendicular to the axonal fibers (Basser, 1995; Wozniak & Lim, 2006). We selected FA as a main index because it measures the degree of water diffusion anisotropy on a scale from zero to one and characterizes WM microstructural abnormalities (Basser & Pierpaoli, 1996). In addition, FA is the most widely used anisotropy measure (O'Donnell & Westin, 2011). We used Philips Extended Workspace (EWS, Release 2.6.3.1; Philips) to analyze DTI data. FA threshold for line tracking was set to 0.2. The maximum angle threshold was 50°. We performed tractography using the two‐regions‐of‐interest (ROIs) approach. In recent years, analyses have been carried out by segmenting CC fibers using a two‐ROIs approach into multiple regions based on the cortical regions that the CC projects to (Huang et al., 2005; Lebel, Caverhill‐Godkewitsch, & Beaulieu, 2010). One of our previous studies applied that technique in mood disorder investigations (Yamada et al., 2015). The first reference ROI was focused on the CC in a midsagittal slice, and secondary ROI was seven separate cortices spanning both sides of the midline (Appendix S1). As seen in Figure 1, callosal fibers were separated into seven segments based on their cortical projection zones. Ordered from front to back, the seven sections were as follows: orbital frontal, anterior frontal, superior frontal, superior parietal, posterior parietal, temporal, and occipital. All ROIs were drawn in accordance with specific anatomical landmarks and guidelines that were followed carefully and consistently for all patients (Figure 2). Same as the previous studies (Brandstack, Kurki, Laalo, Kauko, & Tenovuo, 2016; Huang et al., 2005; Lebel et al., 2010), fibers that were clearly not part of the anatomical connectivity of the tracking were manually removed with exclusion ROIs to include only fibers within the desired tract. Calculation of FA was made by averaging all voxels for each region over the entire tracking. Tractography was performed by one operator (Y.O.). In order to assess validity of ROI procedure, another operator (K.T.) who was blinded subjects' diagnosis, age, gender, and handedness analyzed five subjects in the SZ group and five subjects in the HC group, and interoperator reliabilities for FA values were examined. Intraclass correlation coefficients of FA value of seven segments (orbital frontal, anterior frontal, superior frontal, superior parietal, posterior parietal, temporal, and occipital) were 0.903, 0.956, 0.976, 0.976, 0.726, 0.953, and 0.740, respectively.

### 2.5 Statistics

The differences between the SZ and HC groups in age and *z*‐scores of each neurocognitive test were examined by independent‐samples *t* test. Gender difference between the groups was assessed using the chi‐square test. Using Pearson's correlation test, correlation of FA of each callosal segment with age was assessed in the HC groups, and correlation of FA of each callosal segment with age and equivalent doses of antipsychotics was assessed in the SZ group. Correlation of the *z*‐score of neuropsychological tests with equivalent doses of antipsychotics was also assessed using Pearson's correlation test in the SZ group. In the above analyses, the statistical significance level was set at *p* < 0.05. Stepwise multiple regression analysis was performed, with the *z*‐scores of list learning test, digit sequencing test, token motor test, verbal fluency test, symbol coding test, the Tower of London test, and the composite score as dependent variables, and the FA of callosal fibers in each segment as independent variables to investigate the relationship between the FA and cognitive function in all subjects. The statistical significance level was set at *p* < 0.0071 (adjusted for the Bonferroni correction; 0.05/7 cognitive scores) for cognitive tests. If we find significant relationship between FA of callosal fibers and *z*‐scores of cognitive subtests in all subjects, subsequent stepwise multiple regression analysis in same relation was performed in the SZ group. The statistical significance level was set at *p* < 0.05/number of tests (adjusted for the Bonferroni correction). Stepwise multiple regression analysis was also performed to investigate the relationship between the FA of callosal fibers in each segment and PANSS scores in the SZ group. The statistical significance level was set at *p* < 0.0125 (adjusted for the Bonferroni correction; 0.05/4 PANSS subtests). Independent‐samples *t* test was used to examine differences between the SZ and HC groups in the FA of callosal fiber in the segment which identified the significant relation in regression analysis in the SZ group. The statistical significance level was set at *p* < 0.05/number of tests (adjusted for the Bonferroni correction). In the same approach, stepwise multiple regression analysis was also performed with other DTI index, which includes the ADC, AD, and RD of callosal fibers in each segment as independent variables. All statistical analyses were performed using the IBM SPSS Statistics for Windows (IBM Japan, Ltd.).

## 3 RESULTS

### 3.1 Demographic and clinical characteristics

There were no differences in age and gender between the SZ and HC groups (Table 1). In the BACS, the SZ group showed significantly lower *z*‐scores in six subtests and composite scores when compared to those in the HC groups (Table 1).

### 3.2 Correlation of the FA of callosal fibers with age and equivalent dose of antipsychotics

The FA of callosal fibers significantly correlated with age in the superior frontal segment (*r* = −0.604, *p* = 0.006) and posterior parietal segment (*r* = 0.506, *p* = 0.027) in the HC group and in the anterior frontal segment (*r* = −0.481, *p* = 0.037) in the SZ group. There were no significant correlations between the FA of callosal fibers and equivalent dose of antipsychotics in the SZ group.

### 3.3 Correlation of the *z*‐score of neuropsychological tests and equivalent doses of antipsychotics

There were no significant correlations between the *z*‐score of neuropsychological tests and equivalent dose of antipsychotics in the SZ group (verbal memory, *r* = −0.079, *p* = 0.749; working memory, *r* = 0.238, *p* = 0.326; token motor test, *r* = −0.151, *p* = 0.536; verbal fluency test, *r* = 0.155, *p* = 0.525; attention, *r* = 0.077, *p* = 0.754; executive function, *r* = −0.150, *p* = 0.541; composite score, *r* = −0.017, *p* = 0.943).

### 3.4 Stepwise multiple regression analysis of the FA

**Figure 3:**
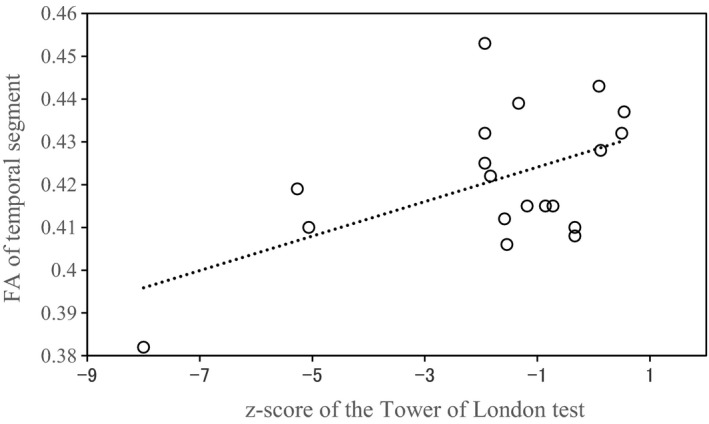
Scattergram for the association between fractional anisotropy (FA) of callosal fibers in the temporal segment and score of Tower of London test in the schizophrenia (SZ) group

In all subjects, the FA of callosal fibers in the temporal segment was significantly associated with the *z*‐scores of token motor test, Tower of London test, and the composite score. The regression for token motor test identified the FA of temporal segment as predictive variable accounting for 19.1% of the variance (*Β* = 46.2; *F* = 8.52; *p* = 0.006). The regression for the Tower of London test identified the FA of temporal segment as predictive variable accounting for 24.0% of the variance (*B* = 58.6; *F* = 11.3; *p* = 0.002). The regression for the composite score identified the FA of temporal segment as predictive variable accounting for 18.5% of the variance (*B* = 38.8; *F* = 8.14; *p* = 0.007). In the SZ group, the FA of callosal fibers in the temporal segment was significantly associated with the *z*‐score of the Tower of London test. The regression for the Tower of London test identified the FA of temporal segment as predictive variable accounting for 29.5% of the variance (*B* = 73.2; *F* = 7.12; *p* = 0.016; Figure 3). In the SZ group, the FA of callosal fibers in the posterior parietal segment was significantly associated with the scores of the PANSS positive, PANSS negative, PANSS general psychopathology, and PANSS total. The FA of callosal fibers in the temporal segment was significantly associated with the score of the PANSS negative. The regression for the PANSS positive identified the FA of posterior parietal segment as predictive variable accounting for 39.8% of the variance (*B* = 275.84; *F* = 11.26; *p* = 0.004). The regression for the PANSS negative identified the FA of posterior parietal segment (*B* = 396.79; *p* < 0.001) and temporal segment (*B* = −197.60; *p* = 0.004) as predictive variable accounting for 65.9% of the variance (*F* = 15.45; *p* < 0.001). The regression for the PANSS general psychopathology identified the FA of posterior parietal segment as predictive variable accounting for 52.4% of the variance (*B* = 567.38; *F* = 18.72; *p* < 0.001). The regression for the PANSS total identified the FA of posterior parietal segment as predictive variable accounting for 54.4% of the variance (*B* = 1,121.63; *F* = 20.26; *p* < 0.001).

### 3.5 Differences in the FA of callosal fiber in the segment which identified the regression in the SZ group between the SZ and HC groups

**Figure 4:**
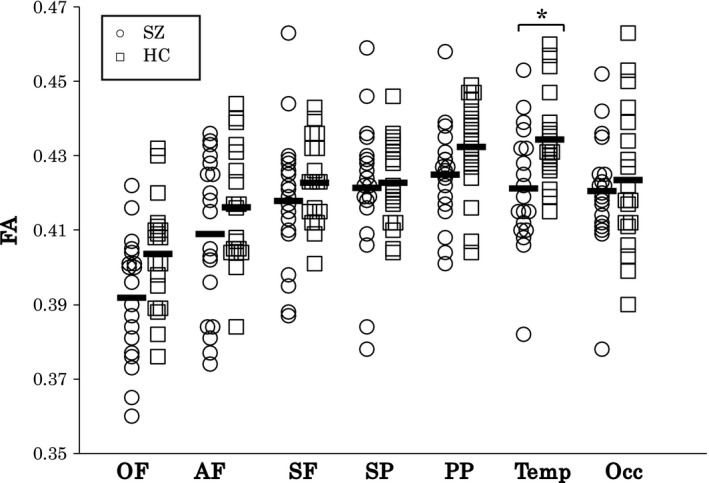
Scattergram for the FA of callosal fibers in the OF, AF, SF, SP, PP, Temp, and Occ segments in HC and SZ groups. The black bars represent means of each segment. Data marked * are significant at *p* < 0.025. AF, anterior frontal; FA, fractional anisotropy; HC, healthy controls; Occ, occipital; OF, orbital frontal; PP, posterior parietal; SF, superior frontal; SP, superior parietal; SZ, schizophrenia; Temp, temporal

Independent‐samples *t* test revealed significant differences in FA between the SZ and HC groups in temporal segment (*t* = 2.80, *p* = 0.008) but not in posterior parietal segments (*t* = 1.75, *p* = 0.088; Figure 4 and Table 2).

**Table 2:** FA and callosal fibers in the OF, AF, SF, SP, PP, Temp, and Occ segments in the HC and SZ groups

	HC group	SZ group	Statistics	
	Mean ± *SD*	Mean ± *SD*	*t*	*p*
OF	0.404 ± 0.015	0.392 ± 0.017		
AF	0.416 ± 0.016	0.409 ± 0.021		
SF	0.423 ± 0.011	0.418 ± 0.019		
SP	0.423 ± 0.011	0.421 ± 0.019		
PP	0.432 ± 0.013	0.425 ± 0.013	1.75	0.088
Temp	0.434 ± 0.012	0.421 ± 0.016	2.80	0.008
Occ	0.423 ± 0.020	0.421 ± 0.015		

Abbreviations: AF, anterior frontal; FA, fractional anisotropy; HC, healthy controls; Occ, occipital; OF, orbital frontal; PP, posterior parietal; *SD*, standard deviation; SF, superior frontal; SP, superior parietal; SZ, schizophrenia; Temp, temporal.

### 3.6 Statistical analysis on the ADC, AD, and RD

In all subjects, the regression for verbal memory identified the ADC of orbital frontal segment (*B* = −24.58; *p* = 0.004) and posterior parietal segment (*B* = 10.88; *p* = 0.02) as predictive variable accounting for 25.5% of the variance (*F* = 6.00; *p* = 0.006). The regression for symbol coding test identified the ADC of posterior parietal segment (*B* = 13.01; *p* = 0.027) and occipital segment (*B* = −19.13; *p* = 0.003) as predictive variable accounting for 26.6% of the variance (*F* = 6.33; *p* = 0.005). The regression for the composite score identified the ADC of orbital frontal segment (*B* = −15.46; *p* = 0.037), posterior parietal segment (*B* = 10.47; *p* = 0.008), and occipital segment (*B* = −9.10; *p* = 0.035) as predictive variable accounting for 34.2% of the variance (*F* = 5.89; *p* = 0.002). In the SZ group, the ADC of callosal fibers in the occipital segment was significantly associated with the *z*‐score of verbal memory and the composite score. The regression for verbal memory identified the ADC of occipital segment as predictive variable accounting for 36.2% of the variance (*B* = −12.98; *F* = 9.64; *p* = 0.006), and the regression for the composite score identified the ADC of occipital segment as predictive variable accounting for 37.4% of the variance (*B* = −11.91; *F* = 10.16; *p* = 0.005).

In all subjects, the regression for symbol coding test identified the AD of temporal segment (*B* = 10.08; *p* = 0.015) and occipital segment (*B* = −13.01; *p* = 0.007) as predictive variable accounting for 25.3% of the variance (*F* = 5.92; *p* = 0.006). The regression for the Tower of London identified the AD of anterior frontal segment (*B* = −12.44; *p* = 0.020) and temporal segment (*B* = 11.55; *p* = 0.003) as predictive variable accounting for 25.7% of the variance (*F* = 6.04; *p* = 0.006). In the SZ group, the AD of callosal fibers in the orbital frontal and occipital segment was significantly associated with the *z*‐score of symbol coding test. The regression for symbol coding test identified the AD of orbital frontal segment (*B* = 28.84; *p* = 0.024) and occipital segment (*B* = −20.66; *p* = 0.001) as predictive variable accounting for 50.5% of the variance (*F* = 8.16; *p* = 0.004).

In all subjects, the regression for verbal memory identified the RD of the orbital frontal segment (*B* = −21.08; *p* = 0.013), the temporal segment (*B* = 13.97; *p* = 0.012), and the occipital segment (*B* = −10.96; *p* = 0.041) as predictive variable accounting for 34.5% of the variance (*F* = 5.96; *p* = 0.002). In the SZ group, the RD of callosal fibers in the occipital segment was significantly associated with the *z*‐score of verbal memory. The regression for verbal memory identified the RD of occipital segment as predictive variable accounting for 33.9% of the variance (*B* = −13.10; *F* = 8.74; *p* = 0.009). In the SZ group, the RD of callosal fibers in the posterior parietal and occipital segment was significantly associated with the scores of the PANSS general psychopathology and PANSS total. The regression for the PANSS general psychopathology identified the RD of posterior parietal segment (*B* = −398.75; *p* = 0.001) and occipital segment (*B* = 109.26; *p* = 0.010) as predictive variable accounting for 54% of the variance (*F* = 9.40; *p* = 0.002). The regression for the PANSS total identified the RD of posterior parietal segment (*B* = −751.67; *p* = 0.001) and occipital segment (*B* = 227.06; *p* = 0.007) as predictive variable accounting for 52.3% of the variance (*F* = 8.78; *p* = 0.003).

In the AD, independent‐samples *t* test revealed significant differences in orbital frontal segment (*t* = −3.06, *p* = 0.004) but not in occipital segment (*t* = −1.05, *p* = 0.300). In the ADC and RD, independent‐samples *t* test revealed no significance.

## 4 DISCUSSION

In the current study, we extracted and divided the CC fibers into seven regions based on the cortical projection regions, and investigated the relationship between the FA values of the CC fibers and cognitive function in schizophrenia. In the SZ group, FA values of the CC that connects the bilateral temporal lobe cortices associated with executive function scores, and FA value of this tract was significantly decreased compared to the HC group. These results indicate an association between microstructural abnormalities of the CC white matter and cognitive dysfunction in schizophrenia.

In the current study, we found a statistically significant association between white matter abnormalities and executive function impairment. Impairment in executive function is one of the most common dysfunctions observed in disease courses of schizophrenia (Orellana & Slachevsky, 2013). We assessed executive function using the Tower of London test. The Tower of London test requires several cognitive processes including working memory (Elliott, 2003), processing speed, response inhibition (Asato, Sweeney, & Luna, 2006; Zook, Davalos, Delosh, & Davis, 2004), and visuospatial processing (Newman, Carpenter, Varma, & Just, 2003), necessitating functional coordination among multiple cortical and subcortical regions (Unterrainer & Owen, 2006). As white matter fibers connect brain regions, many studies have reported a relationship between white matter abnormalities and cognitive function in schizophrenia (Canu, Agosta, & Filippi, 2015). Executive dysfunction of schizophrenia has been reported to be associated with white matter abnormalities in major fiber bundles that connect frontal and temporal lobes, such as superior longitudinal fasciculus and uncinate fasciculus (Kubicki et al., 2002, 2003; Nestor et al., 2004; Pérez‐Iglesias et al., 2010). In schizophrenia, associations also have been reported between superior longitudinal fasciculus and working memory (Karlsgodt et al., 2008), uncinate fasciculus and verbal memory (Nestor et al., 2004; Szeszko et al., 2008), inferior longitudinal and inferior frontooccipital fasciculi and processing speed, verbal learning, and visual learning (Liu et al., 2013), diffuse white matter abnormalities and processing speed (Karbasforoushan, Duffy, Blackford, & Woodward, 2015; Rigucci et al., 2013), visual memory (Rigucci et al., 2013), and social cognition (Rigucci et al., 2013). In the current study, we found the significant association between FA values of the CC fibers connecting bilateral temporal lobe cortices and executive function scores. Studies reported association between bilateral cortical thickness reductions in the temporal lobe and executive dysfunction in schizophrenia (Hartberg et al., 2010), as well as an association between white matter volume reductions in the temporal lobe and verbal memory, attention, problem solving, and working memory dysfunctions in a follow‐up study of early‐onset schizophrenia (Andreasen et al., 2011). The current results suggest that the disconnection between bilateral temporal lobe cortices contributes to poor executive function in schizophrenia.

The SZ group demonstrated decreased FA values in the temporal segments of CC white matter fibers relative to the HC group. Impairment in the temporal lobe with schizophrenia was reported repeatedly. Ellison‐Wright and Bullmore carried out meta‐analysis of the coordinates of fractional anisotropy differences (Ellison‐Wright & Bullmore, 2009). This meta‐analysis of 15 studies (including a total of 407 patients with schizophrenia and 383 comparison subjects) found that significant reductions were present in the left frontal deep white matter and the left temporal deep white matter (Ellison‐Wright & Bullmore, 2009). The second region, in the temporal lobe, is traversed by WM tracts interconnecting the frontal lobe, insula, hippocampus–amygdala, and temporal and occipital lobe. This suggests that WM tracts in the temporal lobe may affect in schizophrenia, with the disconnectivity of the gray matter regions which they link. Decreased FA values of the CC on the DTI whole‐brain analysis in schizophrenia have been repeatedly reported in the rostrum (Ellison‐Wright et al., 2014; Fujino et al., 2014; Gu et al., 2016; Hummer et al., 2016; Kochunov et al., 2014; Kong et al., 2011; Lener et al., 2015; Melicher et al., 2015; Pérez‐Iglesias et al., 2010; Pomarol‐Clotet et al., 2010; Roalf et al., 2013; Spalletta et al., 2015; Zhang et al., 2014, 2016), body (Fujino et al., 2014; Melicher et al., 2015; Pérez‐Iglesias et al., 2010; Roalf et al., 2013; Zhang et al., 2014, 2016), and splenium of the CC (Cheung et al., 2008; Ellison‐Wright et al., 2014; Fujino et al., 2014; Gasparotti et al., 2009; Melicher et al., 2015; Zhang et al., 2014). A meta‐analysis of 22 studies found decreased FA values in the genu and splenium of CC in schizophrenia (Zhuo, Liu, Wang, Tian, & Tang, 2016). These previous studies suggest the validity of segmenting the CC fibers in an anatomically accurate manner when comparing the FA values between patients with schizophrenia and healthy individuals. Several studies have examined decreased FA values in schizophrenia by segmenting the CC in the sagittal slices (Balevich et al., 2015; Knöchel et al., 2012; Li et al., 2014; Rotarska‐Jagiela et al., 2008). Balevich et al. (2015) divided the CC into five anteroposterior segments, but did not find statistically significant decrease of FA values in any specific segments. In studies with segmentation of the CC into nine regions, statistically significant FA reductions were observed in the inferior and superior genu, isthmus (Knöchel et al., 2012), anterior, middle, posterior genu, posterior body, anterior splenium (Li et al., 2014), inferior and superior genu, and splenium (Rotarska‐Jagiela et al., 2008). Whitford et al. parcellated the CC fibers into six segments based on the cortical regions they projected, and examined the difference between patients with schizophrenia and healthy participants (Whitford et al., 2010). They found a statistically significant decrease in FA of the frontal fibers in the patient group. FA values of the temporal fibers were reduced in the schizophrenia group but not statistically significantly so. The discrepancy between the current study and Whitford's study may be partially explained by differences in the DTI methods of analysis and in the study participants. In the current study, we divided the CC fibers based on their cortical projection regions using the two‐ROIs approach. We followed the procedures used in relevant previous studies to determine the location of ROIs and exclusion criteria (Huang et al., 2005; Lebel et al., 2010; Yamada et al., 2015), and we achieved tractography with high anatomical accuracy in each region. On the other hand, Whitford et al. segmented the CC into clusters after whole‐brain tractography. Our study participants included both men and women; however, Whitford's study included only men. TSA is useful as it delineates how fiber tracts connect functional brain regions, providing information on structural connectivity. Our results suggest microstructural abnormalities in the temporal regions of the CC white matter fibers connecting the two hemispheres.

In our SZ group, we observed statistically significant relation between FA values and PANSS scores in the temporal and posterior parietal segments of CC white matter fibers connecting the two hemispheres. The FA of callosal fibers in the temporal segment was significantly negatively associated with the score of the PANSS negative. Several studies have reported that executive function in schizophrenia was associated with the PANSS negative score (Clark, Warman, & Lysaker, 2010; Kishi et al., 2010; Rodriguez‐Jimenez et al., 2010). Microstructural abnormalities of callosal fiber in the temporal segment may be associated with negative symptom in schizophrenia. On the other hand, the FA of callosal fibers in the posterior parietal segment was significantly positively associated with all scores of the PANSS, indicating higher FA related to the higher severity of the symptoms. These results suggest paradoxical effect of anisotropy of callosal fibers in the posterior parietal segment on psychiatric symptom. However, these results should be interpreted cautiously because there was no significant difference in FA of callosal fibers in the posterior parietal segment between the SZ and HC groups. There has been no consensus on the relationship between white matter abnormalities and psychiatric symptoms of schizophrenia. With regard to FA values of the CC and psychiatric symptoms, study results are inconsistent. They reported FA values of the anterior CC and negative correlation with both negative symptoms (Gu et al., 2016; Kubicki et al., 2008; Nakamura et al., 2012) and positive symptoms (Knöchel et al., 2012; Kubicki et al., 2008). FA values of the posterior CC were negatively correlated with negative symptoms (Rigucci et al., 2013), negatively correlated with positive symptoms (Kubicki et al., 2008), but positively correlated with positive symptoms (Rotarska‐Jagiela et al., 2009). FA values of other white matter fibers have also been reported to have inconsistent directions of association, with studies reporting positive correlations with both positive (Andreasen et al., 2011; Chan et al., 2010; Cheung et al., 2011; Choi et al., 2011; Lee et al., 2013; Moriya et al., 2010; Psomiades et al., 2016; Rotarska‐Jagiela et al., 2009, 2008; Seok et al., 2007; Szeszko et al., 2008; Whitford et al., 2010) and negative symptoms (Lener et al., 2015; Mendelsohn, Strous, Bleich, Assaf, & Hendler, 2006; Michael, Calhoun, Pearlson, Baum, & Caprihan, 2008) and negative correlations both with positive (Lee et al., 2013; Skelly et al., 2008) and with negative symptoms (Balevich et al., 2015; Gu et al., 2016; Luck et al., 2011; Mendelsohn et al., 2006; Michael et al., 2008; Moriya et al., 2010; Rigucci et al., 2013; Szeszko et al., 2008; Wolkin et al., 2003). Many studies reported no relationship between FA values and psychiatric symptoms using whole‐brain analyses (Chen et al., 2013; Kyriakopoulos et al., 2009; Kyriakopoulos, Vyas, Barker, Chitnis, & Frangou, 2008; Liu et al., 2013; Melicher et al., 2015; Sugranyes et al., 2012; Wang et al., 2013) and more specific region‐of‐interest analyses including the CC (Li et al., 2014) and other regions (Kawashima et al., 2009; Kitamura et al., 2005; Kitis et al., 2012; Kumra et al., 2004; Price et al., 2008). The mixed findings in past studies regarding FA values and psychiatric symptoms may be partially explained by the differences in the number of participants, severity of the symptoms of the participants, and their treatment histories.

In our SZ group, the AD value of callosal fibers in the orbital frontal segment significantly associated with attention scores, and the AD value of this tract was significantly increased compared to the HC group. Histological information indicates the AD assesses axonal function (Mac Donald, Dikranian, Bayly, Holtzman, & Brody, 2007). Some previous studies reported no significant difference in AD value of the CC between patients with schizophrenia and healthy controls (Hummer et al., 2016; Kochunov et al., 2014; Liu et al., 2013; Spalletta et al., 2015; Whitford et al., 2010). The largest coordinated meta‐analysis on DTI data showed significantly higher AD value in schizophrenia patients compared with healthy controls in the fornix but showed no significant differences in the AD value in the CC (Kelly et al., 2018). To our knowledge, there is no study reported relationship of AD value with cognitive function in schizophrenia. The studies on patients with essential tremor showed positive correlation between the AD value and cognitive function (Julian et al., 2017; Bhalsing et al., 2015). Same as these previous studies on essential tremor, our SZ group showed significant relationship of increased AD with better attention scores, while AD value in the SZ group was significantly increased compared to the HC group. The further studies are needed to interpret this paradoxical relation of AD value with cognitive function in the SZ group.

The current study has some limitations. First, the gender composition of our HC and SZ groups was not fully matched. All participants in the SZ group were taking antipsychotic medications at the time of the MRI examination. Gender ratios were not statistically different between the two groups, and no correlation was found between FA values and antipsychotic medication dosage in the SZ group. However, gender difference and antipsychotic medication use are potential confounding factors for diffusion changes. Second, the sample size was relatively small. Last, no distortion correction was performed in DTI analysis. We offered new insight into relationship between microstructural abnormalities in callosal fibers and cognitive function in schizophrenia, but the results of our study should be confirmed in future studies using more subject with an appropriate control over FA value‐affecting factors.

In summary, we found microstructural abnormalities in the CC white matter fibers connecting bilateral temporal lobe cortices contribute to poor executive function and severe negative symptom in patients with schizophrenia.

## CONFLICT OF INTEREST

None of the authors have potential conflict of interests to be disclosed.

## DATA AVAILABILITY STATEMENT

Research data are not shared.
