# Supplementary material for: Microstructural abnormalities in callosal fibers and their relationship with cognitive function in schizophrenia: A tract‐specific analysis study
Source: Brain Behav. 2019 Jul 8;9(8):e01357. doi: 10.1002/brb3.1357 (PMC6710197; doi:10.1002/brb3.1357)
Supplement: Supplementary file 1 [file BRB3-9-e01357-s001.docx]

***Supplemental method***

Segmentation protocol

The CC in a midsagittal slice is defined as the first reference ROI. The second ROIs were selected seven separate cortices spanning both sides of the midline. Orbital frontal (OF) projection and anterior frontal (AF) projection were identified by delineating the orbital cortex using the coronal slice at the 1/3 of the distance between the anterior edge of the genu of corpus callosum and the most anterior tip of the cerebral hemisphere, and the axial slice at the inferior edge of splenium divided a coronal slice the superior part (AF) and the inferior part (OF). Superior frontal (SF) projection and superior parietal (SP) projection were identified by using the most inferior axial slice which appears the characteristic “Ω” shapes of the central sulcus. SF was anterior to the “Ω” shape, and SP was posterior. Temporal (Temp) projection was identified by setting up the coronal slice at the splenium of CC, where the tapetum are discretely identified. Posterior parietal (PP) projection and occipital (Occ) projection were identified by using the coronal slice at the anterior edge of the parieto-occipital sulcus. The superior part was PP and the inferior was Occ which was divided by the axial slice at the inferior edge of splenium.
